# Penumbral thoughts: Contents of consciousness upon waking

**DOI:** 10.1371/journal.pone.0289654

**Published:** 2023-12-14

**Authors:** Virginia Fedrigo, Matteo M. Galizzi, Rob Jenkins, Jet G. Sanders

**Affiliations:** 1 Department of Psychological and Behavioural Science, London School of Economics and Political Science, London, United Kingdom; 2 Department of Psychology, University of York, York, United Kingdom; Julius-Maximilians-Universität Würzburg, GERMANY

## Abstract

Thoughts shape our experience, choice, and behaviour throughout the day. Yet the content of ‘penumbral thoughts’—first thoughts upon waking—has received very little research attention. Across seven independent samples (total N = 829), we used recall and reflection methods, solicited the same day, to understand what individuals think as they regain consciousness. These penumbral thoughts show remarkable thematic consistency: individuals were most likely to reflect on their somatic or psychological state, focus on temporal orientation, and prioritise waking actions. Survey results demonstrate that temporal and spatial orientation are dominated by the current time and the day ahead, rather than the past or other future timescales. Our results provide some insight into the order of priority in consciousness. We conclude that establishing one’s temporal position is important to the daily process of ‘rebooting’ conscious awareness.

## 1. Introduction

Humans wake up every day. What can we learn from their first waking thoughts? One possibility is that the earliest thoughts reveal levels of priority ascribed to the various constructs which play a role in consciousness. Following the metaphor of a rebooting computer, powering its most essential features (such as working memory) triggers the reboot of secondary features (such as stored memory). Once the system is up and running, new actions can be taken through its interface. When we extend this metaphor to the daily emergence of consciousness, a similar order of prioritisation may take place. Identifying which thoughts occur first allows us to identify which processes receive cognitive priority. Previous studies have shown that experiences of waking up can reverberate for several hours. For example, self-reported anticipation of stress first thing in the morning reduced that day’s working memory [1]. Similarly, a workplace correlational study found that waking up positively left employees more likely to perceive interactions with their customers and work quality more positively too [2]. Such findings demonstrate how early thoughts can set the tone of the rest of the day and may predict variability between thoughts and behaviour over the course of that day.

The first thoughts emerge during a qualitatively different cognitive state from thoughts that emerge during normal wakeful cognition. This state, during the time window between sleeping and being awake, is sometimes known as sleep inertia—“the temporary time of sleepiness, disorientation and impaired cognitive performance experienced upon awakening” [3]. Studies of sleep inertia have emphasised its detrimental effects on performance. Although acute effects of sleep inertia have been shown to dissipate after 15–30 minutes of waking [3], performance on cognitively-challenging tasks during this time has been found to be worse immediately after waking than after 26 hours of sleep deprivation [4]. Similarly, complex planning of military strategy amongst junior officers was found to be impaired immediately after waking [5]. These behavioural findings suggest a relatively basic and primal level of cognition during the transitory period between sleep and wakefulness, devoid of sophisticated levels of thought.

Converging evidence comes from studies which have measured people’s physiological profile during this period. This transition between sleep and wakefulness is marked by a clear sequence of synchronised neurological activity comprising the thalamic nuclei and cingulate cortex [6] marking a large shift in patterns of neural activation. Cognition immediately after waking has been associated with a number of neural correlates, such as increased power of delta waves (the lowest frequency brain waves, associated with the deepest phases of sleep) [7] and decreased blood flow to frontal regions, relative to wakeful activity [8] These neural findings could explain the distinct cognition seen in sleep inertia.

In sum, the work on sleep inertia suggests that thoughts during the transition from sleep to wakefulness may have a distinct profile from thoughts during either full sleep or full wakefulness. We refer to these as *Penumbral Thoughts* by analogy to the boundary between shadow to light. If penumbral thoughts reflect cognitive rebooting, they may be less cognitively sophisticated and less variable across individuals, relative to typical wakeful thought [9, 10]. To the best of our knowledge, however, no previous study has examined penumbral thoughts from a psychological science perspective. This omission is perhaps surprising, given 1) the ubiquity of regaining consciousness as a daily experience, 2) it is frequently studied in other disciplines i.e., through literary or cinematic representation (as in Proust’s ‘Swann’s Way’ [11], discussed in [12], or Charles Dickens’ ‘A Christmas Carol’ [13], discussed in [14]), and 3) the insights gleaned from behavioural and physiological studies of sleep inertia. It reveals a gap in understanding between the content of thoughts during sleep (i.e., dreaming; [15, 16]) [9, 17–22], and the content of thoughts during full wakefulness—both of which have been studied intensively in psychological research.

The contrast with full wakefulness is particularly relevant here. Over the last decade, several studies have sought to characterise the content of wakeful thought, including its temporal and spatial orientation, protagonist focus, and affective valence [9, 17–22]. For example, thought content is only on-task (in the present) about half of the time [9]. The other half is focused on the past (episodic memory) or planning of the future (episodic foresight) [9, 22]. Estimates suggest that about two-thirds of wakeful thoughts are future-oriented, and one-third are past-oriented [17, 18]. D’Argembeau, Renaud & van der Linden found that on a typical day, an average 42.5% of thoughts were future-oriented, with 31% of these future-oriented thoughts pertaining to later in the same day [18]. Other studies have found that the content of thought is often related to oneself, with the affective valence more often negative or neutral than positive [17, 20, 21]. These regularities matter, not least because they can affect the thinker’s mood: future-orientation and positive mood tend to go together; and past-orientation and negative mood tend to go together [19]. These associations suggest that the temporal orientation of penumbral thoughts may shape the quality of conscious experience for the day ahead.

In the current study, we set out to categorise the content, valence, protagonist, and orientation of penumbral thoughts. To monitor the consistency of penumbral thoughts, we collected data in seven independent samples, one on each day of the week [23, 24]. If penumbral thoughts reflect early prioritisation, we expect a degree of consistency across samples and person demographics, such that a small number of readily identifiable themes emerge. We also expect future orientation with a short horizon, geared towards ordering behaviour over the day ahead.

## 2. Methods

### 2.1. Participants

A total of 829 paid participants from the UK were recruited on the Prolific platform over two weeks in November 2020 (117–121 participants total per day of week; mean age = 32.7 years, age range = 18–75; 71.5% female). See Supplementary Material 1 in S1 File for the age and gender distribution per sample. All participants provided informed consent (University Ethics approval number 07564).

### 2.2. Materials and procedure

The experiment was run entirely online using Prolific. The survey was created and compiled using Qualtrics, which was used to administer task instructions, present test materials, and record participants’ responses. Participants accessed the experiment from their own devices.

#### 2.2.1. Open recall question

Participants were first asked to complete a free recall question, “*What was your first thought when you woke up this morning*?”, with waking up described as “*the first moment of consciousness after sleep*” in order to clarify the intended period of time. We opted to ask for the first thought as a thought could also refer to a direct thought process (i.e., where am I?), but also an experience (i.e. It’s cold here) or emotional state (‘I am tired’). The intention here was to capture information that participants volunteered when they were not led to any particular theme.

#### 2.2.2. Reflections

Reflection items were used to elicit data on specific topics of interest. Participants were asked whether they (i) already knew, or (ii) sought to establish the time, day, or place when they first woke up (See Table 1A). We compared knowing or establishing of temporal information (time or day; which is typically different on successive wakings and therefore may be less known) to the baseline of spatial information (place; which is typically the same on successive wakings and therefore more known (1)). Participants responded on a Likert scale from Never (0), to Sometimes (1), to Usually (2) to Always (3). For example: *How often does the following statement apply*: *When I wake up*, *I want to establish the time*.

**Table 1:** Statements on A) prior knowledge and B) temporal orientation were presented to participants. Participants responded on a Likert scale from Always (3), Usually (2), Sometimes (1), to Never (0).

**A) Prior knowledge**		
When I wake up, …		
**Prior knowledge**	**…I know…**	**…I want to establish…**
**Place**	. . .the place	…the place
**Day**	…the day	…the day
**Time**	…the time	…the time
**B) Temporal orientation**		
As I wake up, I think about…		
**Direction**	**Future**	**Past**
**Distance (day)**	…the day ahead	…the previous day
**Distance (week)**	…the week ahead	…the previous week
**Distance (year)**	…the year ahead	…the previous year

Next, participants were asked to reflect on an additional six statements to establish the temporal orientation of their penumbral thoughts. Our aim was to distinguish between temporal direction (i.e., future or past) and temporal distance (day, week, or year; see Table 1B). For example: *How often does the following statement apply*: *As I wake up*, *I think about the day ahead*.

### 2.3. Analysis

#### 2.3.1. Open recall responses

*2*.*3*.*1*.*1*. *Thought characterisation*. The purpose of thought characterisation is to capture the variety of unprompted thoughts and rank the most commonly occurring themes [25]. To identify common themes of penumbral thought content we used a blended approach between open and template coding [26, 27] and an intercoder reliability procedure to develop a codebook [28]. Once themes had been identified, we used a co-occurrence analysis between the three identified themes and age, gender, and weekday. See Supplementary Materials 2 in S1 File for full methodology, decision log, finalised codebook, and analysis. In line with the procedures in qualitative coding [29], no inferential statistics is used, but rather a focus is drawn to ranking between classes of response [25].

*2*.*3*.*1*.*2*. *Thought context*. To establish the context of penumbral thoughts each response was also rated on a number of dimensions previously identified in wakeful thought content [9, 19]. These include temporal orientation (past, present, future), protagonist (self or other), and valence of thought (positive, neutral, negative). As we were interested in the information that people seek to acquire when emerging from sleep, we also analysed sentence formulation (question or statement). Binomial tests with Clopper-Pearson 95% CI, controlled for multiple comparisons, were used to test for differences between reports across categories. To assess differences across age, gender, and weekdays we used chi-square tests across each dimension, also controlled for multiple comparisons.

#### 2.3.2. Reflection responses

To show how often (DV) participants knew or sought to establish (IV1) thought about time, day, or place (IV2) when they first woke up, we used a 2x3 repeated measures ANOVA. Similarly, we used a 2x3 repeated measures ANOVA of day, week, year (temporal distance, IV1) by its temporal direction (past or future, IV2) to establish the temporal orientation of participants’ firsts (DV). Next, we ran a 2x3x7 mixed design ANOVA, repeated the above two analyses adding in the factor of the day of the week (IV3), to measure consistency in any observed patterns across weekdays.

## 3. Results

### 3.1. Open responses

97.8% of participants reported thought content (811 of 829). The remaining 18 participants left the response box blank (n = 11), mentioned that they had forgotten (n = 6) or reported ‘nothing’ (n = 1).

### 3.2 Thought characterisation

**Fig 1:**
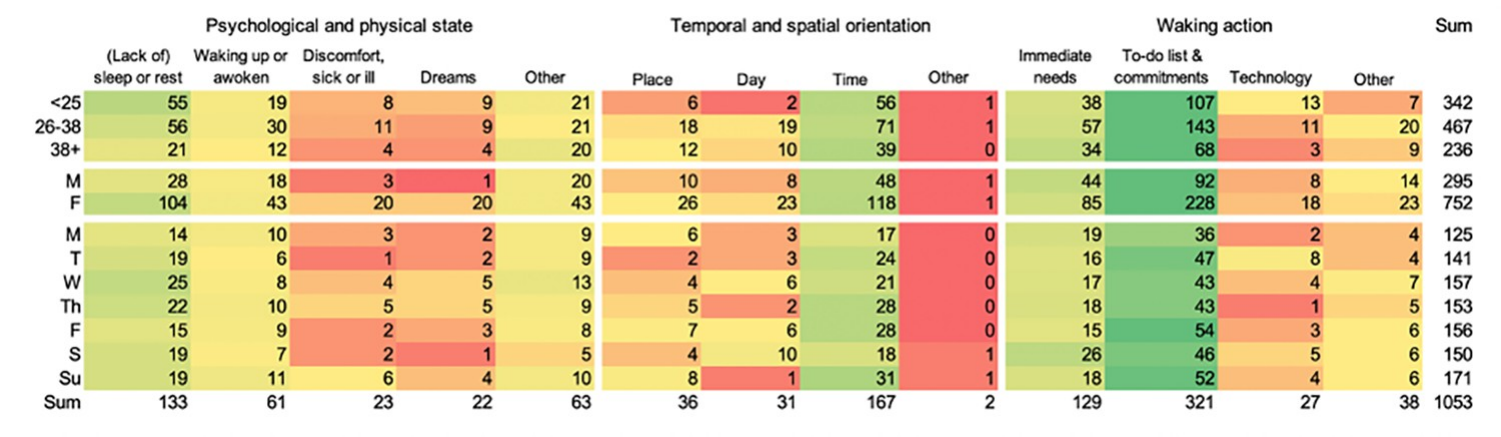
Counts of codes based on free response statements to the question *“What did you think about when you first woke up*?” by demographic groups (age and gender) and by days of the week.

Three themes of penumbral thought content were identified: 1) reflection on present psychological or physical state (including transition from sleep to wakefulness), 2) temporal and/or spatial orientation, 3) establishing waking action (see Fig 1 for selective codes). Below we describe the themes and codes which elicited at least 3% of participants responses, in its ranked order.

#### 3.2.1. Description of themes

A total of 1053 codes were awarded (range: 0–8 codes per response) to 807 responses (99.5% of all responses). See Fig 1 for a distribution of counts across themes. Due to imbalance in gender and age groups, comparisons are made across rows. Counts of codes in each theme group can be found in Supplementary Materials 3 in S1 File.

*3*.*2*.*1*.*1*. *Reflection on psychological and physical state*. For 1 out of 4 participants, their penumbral thought referred to their own psychological or physical state. Most of these referred to the transition from sleep to wakefulness. Of these participants, a quarter referred to their sleep or to still being asleep (‘*I’ve slept too long*’), another quarter mentioned (still) being tired (and needing more sleep). Others described the waking up process (‘*disoriented and tired’*) or specifically what they were awoken by (such as an alarm or an interruption *‘oh no baby is crying’*). A few participants mentioned feelings of physical discomfort (‘*headache’*, *‘feel ill’*).

*3*.*2*.*1*.*2*. *Temporal and/or spatial orientation*. 1 in 4 participants aimed to establish the time, day, or place when they first woke up. 1 in 5 aim to establish the time and, 1 in 10 participants note their exact penumbral thought to be ‘*What time is it*?*’*. Some others aimed to establish ‘*What day it is*’ or how they expected to fill their time that day (‘*what do I need to do today*’). Some participants referred to spatial orientation. Most frequently they mentioned elements of change in their surroundings, such as the weather (‘*is it snowing*?*’*, *‘what is the weather like*?*’*).

*3*.*2*.*1*.*3*. *Establishing waking action*. 1 in 2 described thinking about the actions they needed to take that day. These included immediate bodily needs (*‘I need to eat’*, *‘need the bathroom’*), but could also refer to longer timeframes of action. 1 in 3 participants referred to (items on) their ‘to-do list’ for the day, by noting this as a question (‘*what meetings do I have today*?’) or listing tasks for the day explicitly (*‘need to do my exercises’*). None of the participants explicitly described tasks further than a day ahead.

#### 3.2.2. Consistency across age, gender, and weekday

To test for consistency of thought content, we segmented the data by the person characteristics. Co-occurrence analysis of the three emergent themes across three age categories (<25, 25–38 and 38+), gender, and weekday demonstrated high levels of consistency. Only two statistically significant associations were found. First, participants under the age of 25 were more likely to report physical and psychological state upon waking (OR: 1.40 (95% CI 1.00–1.95)). A qualitative inference suggests that this may be driven by fewer young participants describing being awoken (possibly due to lack of childcare responsibilities). Second, across weekdays, reports of time, day or place were more likely on Mondays than on other days (OR: 1.73 (95% CI 0.97–2.99)), and less likely on Sundays than on other days (OR: 0.50 (0.23–0.96)). See Table 2 for details.

**Table 2:** Co-occurrence between themes, age, gender, and weekday presented in odds ratio, 95% confidence interval. *p < 0.05.

	Physical and Psychological State	Temporal and Spatial Orientation	Waking Action
**Age**			
under 25	1.40 (1.00–1.95)*	0.84 (0.52–1.33)	1.04 (0.73–1.47)
26–38	0.73 (0.52–1.02)	1.20 (0.76–1.95)	0.95 (0.67–1.35)
over 38	0.87 (0.60–1.26)	1.37 (0.85–2.17)	1.05 (0.71–1.52)
**Gender**			
Female	Reference		
Male	0.99 (0.69–1.40)	1.03 (0.63–1.63)	1.20 (0.83–1.71)
**Weekday**			
Monday	1.11 (0.69–1.76)	1.73 (0.97–2.99)*	0.94 (0.56–1.53)
Tuesday	0.75 (0.45–1.21)	0.46 (0.19–0.99)	1.27 (0.79–2.01)
Wednesday	1.10 (0.70–1.71)	0.78 (0.39–1.47)	1.06 (0.66–1.68)
Thursday	1.34 (0.85–2.08)	1.27 (0.69–2.24)	0.85 (0.51–1.38)
Friday	0.86 (0.53–1.37)	1.37 (0.75–2.39)	0.85 (0.51–1.38)
Saturday	0.82 (0.50–1.31)	1.37 (0.75–2.39)	1.06 (0.65–1.69)
Sunday	1.09 (0.71–1.64)	0.50 (0.23–0.96)*	0.99 (0.64–1.53)

### 3.3. Thought context

To characterise the context of penumbral thoughts, we categorised each response on four dimensions: (i) its temporal orientation (past, future), (ii) the protagonist or personal referent (self, other), (iii) its affective valence (positive, negative), and (iv) sentence formulation (statement, question). To avoid confusion between the participant-generated thought content and the above qualities of the thought, we will use the term ‘thought context’ for this group of features. To control for multiple comparisons, a Bonferroni adjusted p-value of 0.0125 was used for statistical inferences.

#### 3.3.1. Distribution of responses across dimensions

**Fig 2:**
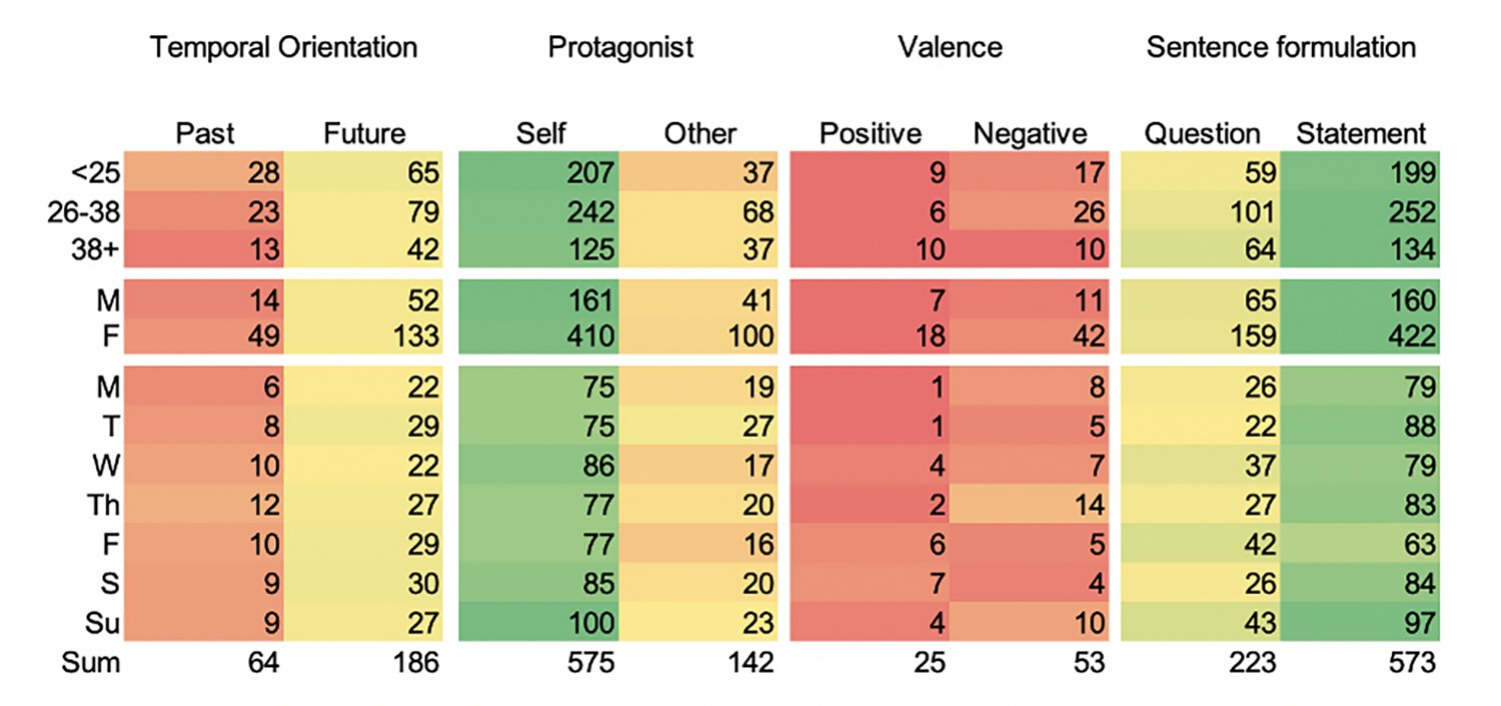
Raw counts of observer ratings of free response statements to the question *“what did you think about when you first woke up*?” based on four dimensions: Temporal orientation, the protagonist, valence, and sentence formulation, by demographic groups (age and gender) and by days of the week.

See Fig 2 for the distribution of responses across dimensions. Due to imbalance in gender and age groups, comparisons are made across rows.

*3*.*3*.*1*.*1*. *Dimension 1*: *Temporal orientation*. Temporal orientation (past, future) was identifiable for 30.2% of the responses (N = 250). Other responses were excluded from further analysis. Of those given a temporal orientation, significantly more (74.4%; N = 186) referred to future events (*‘working today’* and *‘I need to get up as expecting a delivery early’*), than events in the past (N = 64; 25.6%;*‘about the dream I just had’ ‘I messed up salary negotiation during a call with HR for a job i (sic) was interviewing for’*) [p < 0.001, z = -7.65, 95% CI = [68.5%, 79.7%]].

*3*.*3*.*1*.*2*. *Dimension 2*: *Protagonist*. More participants (80.2%; N = 575) expressed having a self-referred thought when they first woke up, than having an other-referred thought (19.8%; N = 142); [p < 0.01, z = 16.13, 95% CI = [77.1%, 83.1%]]. Individually centred statements included reflections on one’s current state (*‘am alive’*, *‘im (sic) still tired’*), a plan for future activities (*‘I’ll go out for a walk’*, *‘check my phone’)*, or personal care needs (*‘I need a wee*.*’*, *‘I need a coffee’)*. Other-centred thoughts referred to members of a social circle (friends, family, or pets on occasion). Qualitative inference indicated that other-centred thoughts were often paired with responsibilities, such as school preparation or other caring responsibilities (‘*get kids ready for school’*, *‘ringing and waking my boyfriend’*, *‘how is my daughter’*). At times, participants mentioned having been awoken by someone or something in their household (*‘oh no baby is crying’*).

*3*.*3*.*1*.*3*. *Dimension 3*: *Valence*. Explicit emotional valence of thought (negative, positive) could be attributed to only 9.4% (N = 78) of responses. Other responses did not express explicit valence, were interpreted as neutral (‘*food*’, ‘*Packaging some parcels’*), and thus excluded from this comparison. Negative statements (67.9%; N = 53 *‘About work*. *I have stressful deadlines today*.*’*) were twice as likely as positive statements (32.1%; N = 25; ‴*Yes*!!!!!*"—I always wake up like this*.*’*); p < 0.001, z = 3.06, 95% CI = [56.4%, 78.1%]. Statements of a negative valence most often referred to feelings of physical discomfort (*‘can’t breathe’*). Positive statements varied and referred to feelings of gratitude (*‘Thank god it’s Saturday*’), or general observations (‘*That I had a good night’s sleep’*, *‘I’m (sic) so happy’)*.

*3*.*3*.*1*.*4*. *Dimension 4*: *Sentence formulation*. More (72.3%; N = 586) penumbral thoughts were formulated as statements than as questions (27.7%; N = 225); [p < 0.001, z = 12.64, 95% CI = [69.0%, 75.3%]. Interestingly, the observed frequency of questions in this sample (N = 225, 27.7%) was significantly higher than the expected frequency based on analyses of everyday speech (5%) [23] [p < 0.001, z = 29.64, 95% CI = [24.7%, 31.0%]]. Common formulations include *‘What are the kids doing*?*’*, *‘Did I oversleep*?*’*. Unsurprisingly this code frequently co-occurs with establishing time or day ‘*What time is it*?*’*; c-coefficient = 0.442).

### 3.4. Consistency across demographics and weekday

Next, we examined the consistency in thought context across person characteristics and the samples for each day of the week. Controlling for multiple comparisons with a Bonferroni correction at a p-value threshold of 0.004, we found no differences were observed between any person characteristics or weekday across any of the dimensions (across age groups: (temporal orientation: [χ2 (2, *N* = 250) = 1.60, *p* = 0.449]; protagonist: [χ2 (2, *N* = 716) = 5.13, *p* = 0.077]; valence: [χ2 (2, *N* = 78) = 5.64, *p* = 0.060]; sentence formulation [χ2 (2, *N* = 809) = 5.27, *p* = 0.072]); between genders: (temporal orientation: [*X*^*2*^ (1, *N* = 248) = 0.83, *p* = 0.361]; protagonist [*X*^*2*^ (1, *N* = 712) = 0.04, *p* = 0.835]; valence:[*X*^*2*^ (1, *N* = 78) = 0.50, *p* = 0.478]; sentence formulation [*X*^*2*^ (1, *N* = 806) = 0.29, *p* = 0.588)]); across weekdays: (temporal orientation: [χ2(6, N = 811) = 8.165, p = 0.226]; protagonist:[χ2(6, N = 811) = 4.139, p = 0.658]; sentence formulation: [χ2(6, N = 811) = 14.609, p = 0.024])). We conclude that there are high levels of consistency in thought context.

### 3.5. Reflection responses

**Fig 3:**
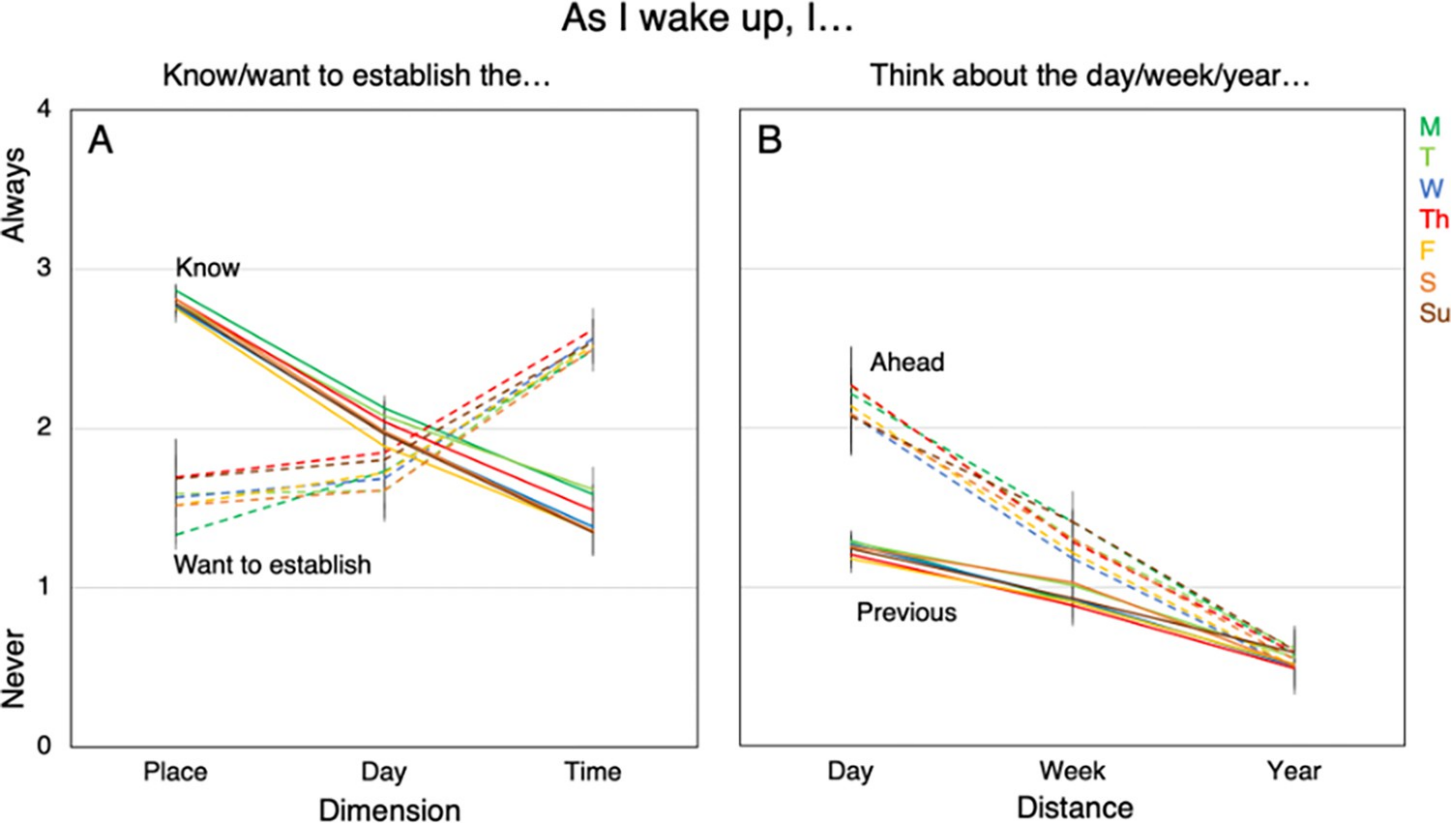
How often participants (**A**) know or want to establish the spatial (place) or two temporal (day and time) dimensions and (**B**) have thoughts about the week, day, or year ahead or behind, across seven independent samples (one for each weekday), when they first wake up. Error bars display 95% confidence intervals.

Responses to the rating items are summarised in Fig 3.

#### 3.5.1. Knowing and establishing information

A 2x3 repeated measures ANOVA was performed of prior knowledge (know versus want to establish) by dimension (place, day, time), with knowing place (M = 2.80, SE = 0.02, 95% CI = [2.77, 2.83]), knowing day (M = 2.01, SE = 0.02, 95% CI = [1.96, 2.06]), knowing time (M = 1.45, SE = 0.03, 95% CI = [1.39, 1.51]), wanting to establish place (M = 1.56, SE = 0.05, 95% CI = [1.47, 1.65]), wanting to establish day (M = 1.71, SE = 0.04, 95% CI = [1.64, 1.78]), and wanting to establish time (M = 2.54, SE = 0.03, 95% CI = [2.49, 2.59]). We observed a main effect of prior knowledge [F(1, 821) = 19.90, p < 0.001, η^2^_p_ = 0.024, 95% CI = [0.007, 0.048]], and dimension [F(2, 1642) = 86.60, p < 0.001, η^2^_p_ = 0.095, 95% CI = [0.070, 0.122]]. We also observe a significant interaction between dimension and knowledge [F(2, 1642) = 985.90, p < 0.001, η^2^_p_ = 0.546, 95% CI = [0.516, 0.582]]. Post-hoc comparisons with a Bonferroni correction showed the interaction effect was driven by stark differences between spatial and temporal dimension in terms of prior knowledge and interest in establishing knowledge. There is a need for establishing the time, and a clear lack of knowing the time at the point of waking [pairwise comparison for establishing and knowing time: t = 27.01, p < 0.001, Cohen’s d = 1.385, 95% CI = [1.277, 1.492].

However, there is a strong existing knowledge of place and a weaker inclination towards establishing it [t = -24.6, p < 0.001, Cohen’s d = -1.277, 95% CI = [-1.375, -1.078]]. Lastly, there is a smaller gap between knowing and wanting to establish the day [t = -6.91, p < 0.001, Cohen’s d = -0.339, 95% CI = [-0.476, -0.202]]. The general variation of time in an individual’s waking up and the relative stability in the place help to contextualise these findings. See Supplementary Materials 4 in S1 File for all pairwise comparisons.

To review the heterogeneity of this pattern across weekday, we ran a 2x3x7 mixed design ANOVA of repeated measures orientation of prior knowledge (knowing versus wanting to establish), and dimension of thought (place, day, time) and independent measure of weekday (Monday, Tuesday, Wednesday, Thursday, Friday, Saturday, Sunday). There was a significant main effect of dimension [F(2,1628) = 6.59, p = 0.001, η^2^_p_ = 0.008, 95% CI = [0.001, 0.018]] but no significant main effect of prior knowledge [F(1, 814) = 0.145, p = 0.703, η^2^_p_ = 0.000, 95% CI = [0.000, 0.006]]. Importantly, there was no significant main effect of weekday [F(7,814) = 1.23, p = 0.285, η^2^_p_ = 0.010, 95% CI = [0.000, 0.019]]. There were also no significant interaction effects with weekday [dimension x weekday: F(14, 1628) = 1.04, p = 0.409, η^2^_p_ = 0.009, 95% CI = [0.000, 0.010], prior knowledge x weekday: F(7, 814) = 1.70, p = 0.105, η^2^_p_ = 0.014, 95% CI = [0.000, 0.026], dimension x prior knowledge x weekday: F(14, 1628) = 0.437, p = 0.963, η^2^_p_ = 0.004, 95% CI = [0.000, 7.38e-4]]. This demonstrates that prior knowledge and dimension are consistent across weekdays (see Fig 3A).

#### 3.5.2. Temporal orientation

A 2x3 repeated measures ANOVA of temporal orientation (future versus past) and temporal distance (day, week, year) [day ahead: M = 2.16, SE = 0.03, 95% CI = [2.11, 2.21], week ahead: M = 1.30, SE = 0.23, 95% CI = [1.25, 1.35], year ahead: M = 0.56, SE = 0.02, 95% CI = [0.51, 0.61], day before: M = 1.25, SE = 0.03, 95% CI = [1.20, 1.30], week before: M = 0.94, SE = 0.02, 95% CI = [0.94, 0.94], year before: M = 0.56, SE = 0.02, 95% CI = [0.51, 0.61]] showed a significant main effect of temporal distance [F(2, 1640) = 1632, p < 0.001, η^2^_p_ = 0.666, 95% CI = [0.643, 0.689]], a significant main effect of temporal orientation [F(1,820) = 447, p < 0.001, η^2^_p_ = 0.353, 95% CI = [0.304, 0.399]] and a significant interaction effect for temporal distance and temporal orientation [F(2, 1640) = 391, p < 0.001; η^2^_p_ = 0.306, 95% CI = [0.270, 0.339]]. A follow up analysis, using post-hoc comparisons using a Bonferroni correction revealed that the interaction was driven by an increased focus on the day ahead (relative to the day behind), with smaller differences between week ahead and week behind [t = 12.65, p = < 0.001, Cohen’s d = 0.494, 95% CI = [0.356, 0.633]], and no differences between the year ahead and year behind [t = 1.69, p = 0.536, Cohen’s d = -0.055, 95% CI = [-0.191, 0.081]].

Interestingly, these results also provide some insight into the mental representation of temporal distance and orientation. For example, post-hoc comparisons demonstrate no significant difference in the amount of time thought about the day before and week ahead [t = -1.40, p = 0.727, Cohen’s d = -0.066, 95% CI = [-0.163, -0.030]]. This suggests that these two concepts may be psychologically similar, despite being chronologically very different (one day versus seven days). See Supplementary Materials 5 in S1 File for details.

To analyse the heterogeneity of this pattern across the days of the week, we ran a 2x3x7 mixed measures ANOVA, of repeated measures orientation of thought (future versus past), and temporal distance of thought (day, week, year) and independent measure of weekday (Monday, Tuesday, Wednesday, Thursday, Friday, Saturday, Sunday). Results replicate a significant main effect of temporal distance [F(2, 1626) = 129.92, p < 0.001, η^2^_p_ = 0.138, 95% CI = [0.108, 0.168]], a significant main effect of temporal orientation [F(1, 813) = 15.55, p < 0.001, η^2^_p_ = 0.019, 95% CI = [0.005, 0.041]], and a significant interaction effect between temporal orientation and temporal distance [F(2, 1626) = 15.31, p < 0.001; η^2^_p_ = 0.018, 95% CI = [0.007, 0.033]], but show no significant effect of weekday [F(7, 813) = 0 .98, p = 0.441, η^2^_p_ = 0.008, 95% CI = [0.00, 0.015]], and no significant interaction effects with weekday [temporal distance and weekday: F(14, 1626) = 0.985, p = 0.466, η^2^_p_ = 0.008, 95% CI = [0.00, 0.016]]; temporal orientation x weekday: F(7, 813) = 1.79, p = 0.085, η^2^_p_ = 0.015, 95% CI = [0.00, 0.023]; temporal distance x temporal orientation x weekday: F(14, 1626) = 1.473, p = 0.113, η^2^_p_ = 0.013, 95% CI = [0.00, 0.016]]. In sum, we demonstrate a high level of consistency in temporal orientation and distance of penumbral thoughts across the seven weekdays (see Fig 3B).

## 4. Discussion

In this paper we sought to understand penumbral thoughts—the contents of consciousness at the boundary between sleep and wakefulness. The combination of qualitative and quantitative data reveals a cohesive picture of thought content across age groups, genders, and weekdays. The homogeneity of responses across a broad participant sample suggests that certain cognitive priorities may be characteristic of regaining consciousness. First, we observe a much higher incidence of questions in reports of penumbral thoughts (27%) than expected based on language use elsewhere (5%) [30]. The apparent overrepresentation of questions suggests an orientation towards information seeking. Second, we identify the principal themes of penumbral thoughts—a mental or physical check-in, locating oneself in time, and previewing tasks for the day ahead. Third, we find that temporal location is less well known than spatial location upon waking, and that resolving time and day is a priority.

How do penumbral thoughts compare to other wakeful thoughts? There are some points of contact and some points of departure. Compared with previous studies, we see broadly similar patterns for affective valence [17, 20], self-orientation [17, 20, 31–33], and temporal direction (past vs. future) [17, 18]. On the other hand, penumbral thoughts seem to be especially focused on the short-term future, particularly the day ahead. Whereas D’Argembeau, Renaud & van der Linden found that only about 1 in 3 future-oriented thoughts pertain to the same day [18], we find that nearly all penumbral thoughts concern this timeframe. We also find that time is often the subject of penumbral thoughts, with 1 in 5 participants trying to establish the time as they wake up.

Indeed, the dominance of a small number of themes among penumbral thoughts—internal state, orientation in time, tasks for the day ahead—suggest that the first moments of wakefulness may be especially convergent. Dreams are highly diverse across individuals [15, 16]. Wakeful thoughts are highly diverse across individuals [34–39]. In comparison, penumbral thoughts appear to be much more restricted.

As well as illuminating the nature of penumbral thoughts, our findings contribute to a growing psychological literature on the influence of the weekly cycle. It is well established that mood changes through the weekly cycle, both at the level of mental representations [23] and at the level of reported experience [24, 40]. What is less clear is how these two levels may be related. Early retrieval of the day of the week suggests a path by which stereotypical weekday associations could set the tone for the rest of the day. At the same time, the uniformity of retrieval over the cycle suggests that differential associations for each day will land with similar force.

We note several possible limitations of our study. First, data collection took place during the COVID-19 lockdown period in the UK in November 2020. Given that some people reported difficulties keeping track of time during COVID-19 lockdowns [41], it is possible that our sample captures an unusual level of temporal disorientation. However, if lockdown was dominating participants’ thoughts, we might expect it to be mentioned in their responses. In fact, such mentions were rare (two mention lockdown, two mention COVID-19). Nonetheless, we are aware that the government restrictions could have homogenised the set of experiences across individuals. Generalisability across data collection conditions could be estimated by repeating the study when lockdown restrictions are lifted.

A second limitation is that penumbral thoughts were solicited later in the day through recollection rather than immediately after they occurred. The delay between occurrence and reporting places a considerable burden on recall, with risk of introducing noise into the data. Importantly, the validity of such a method has been well-reported through the use of the day reconstruction method (DRM) [42] and the use of the present data collection method is widespread (e.g., [43–46]). A well-known limitation of the DRM i*s* that delay can hinder recall performance [47]. This may apply more so when individuals rely on logical deductions or perhaps when sleep inertia applies, as could be the case in this study. Future studies could complement this methodology by adapting the sleep diary method to capture penumbral thoughts soon after they arise [48]. Such in-the-moment solicitation could then simultaneously serve to validate the use of DRM for penumbral thought elicitation. We situate this existing research as a first exploration of penumbral thoughts, laying theoretical groundwork for exciting future studies utilising a variety of methods.

Further, as specific question phrasing affects content of dream reports [49], another opportunity for future research is to elicit responses in early waking with different questions. For example, one could ask specifically about emotions, thoughts, and perception at the point of waking. Similarly, within-subject data on penumbral thoughts and daytime thoughts could be collected, further elucidating the differences between states but within an individual (and their specific context).

Future research could also integrate these self-reported findings with measures of neural activity to further characterise the relationship between the penumbral thoughts experienced and the neural correlates of these thoughts, especially in light of established patterns of activation within this sleep to wakefulness state transition [6]. Another interesting avenue for future research would be to examine penumbral thoughts when regaining consciousness in other situations, such as emerging from anaesthesia [50, 51]. Such comparisons would allow us to test whether penumbral thoughts depend on the conditions in which consciousness was lost.

It is also important to note that many of the shared orientations and propensity towards the self in wakefulness thought are known to be affected by shared cultural cognitions (i.e., [52]) and particularly a culturally rooted understanding of self versus others [53]. An interesting future research pursuit would be to investigate the extent to which this applies to penumbral thoughts too.

For now, we offer a first insight into what people think about when they wake up. Across seven independent samples, we find that individuals are most likely to check in on their somatic or psychological state, focus on temporal orientation, and preview waking actions. We conclude that these themes reflect cognitive priorities as waking consciousness reboots.

## Supporting information

S1 File (DOCX)
