# Supplementary material for: Penumbral thoughts: Contents of consciousness upon waking
Source: PLoS One. 2023 Dec 14;18(12):e0289654. doi: 10.1371/journal.pone.0289654 (PMC10721006; doi:10.1371/journal.pone.0289654)
Supplement: S1 File — (DOCX) [file pone.0289654.s001.docx]

**10. Supplementary Materials**

**S1: Demographics by weekday**

| **Day of Week** | **N** | **% Male** | **Average Age** | **Age range** |
| --- | --- | --- | --- | --- |
| **Monday** | **118** | **29.66** | **33.73** | **18-66** |
| **Tuesday** | **117** | **25.64** | **32.61** | **18-66** |
| **Wednesday** | **120** | **32.50** | **31.65** | **18-63** |
| **Thursday** | **117** | **23.08** | **33.34** | **18-67** |
| **Friday** | **118** | **24.58** | **33.69** | **18-75** |
| **Saturday** | **118** | **27.97** | **32.53** | **18-75** |
| **Sunday** | **121** | **33.06** | **31.62** | **18-65** |

**S2: Full methodology of thematic analysis, decision log and codebook**

To identify common themes of penumbral thought content we used a blended approach between open and template coding [1,2]. It is important to note that several codes (temporal content, protagonist, valence, sentence formulation) were templates, whereas the others emerged through open coding. The codes and process were refined through an intercoder reliability procedure [3]. A random 10% of items were coded independently by authors JGS and VF. Once completed, coders discussed emerging codes, and areas of divergence until they reached agreement (see the decision log, Stage 1 below). This yielded a first intercoder rating (Krippendorf’s alpha = 0.855). Following this process, themes originating from the open coding were further distilled to axial codes and resulted in selective codes [4,5].

Next, all items were coded by author VF. To establish a final interrater agreement, score 10% of coded items were randomly selected and coded by author JGS independently. Coders discussed differences in interpretation and agreements were logged (see the decision log, Stage 2 below; Krippendorf’s alpha = 0.954). The coded data was then adjusted in line with the decision log by author VF. Finally, some codes were collapsed into selective codes to simplify the data (see Supplementary Material 3 for the finalised codebook).

In line with the procedures in qualitative coding, no inferential statistics is used, but rather a focus is drawn to ranking between classes of response [6]. This is in line with reporting standards for qualitative research as “cannot be usefully quantified given the nature, composition and size of the sample group, and ultimately the epistemological aim of the methodology” [7]. We do review co-occurrence between the three identified themes for each demographic characteristics of age and gender and across the seven weekdays. Sub-themes could not be analysed due to a minimum count of 20 items per cell. As we had an imbalance in the sample for age and gender, we compared the number of reported accounts for each theme, relative to the number of reports on other themes (by row), controlling for the number of participants in each demographic cell (columns). Where there were more than two cells per comparison, counts were compared with the average count across the others within the same characteristic (age, gender, or weekday), for example: under 25 years old, versus average of 25-38 and over 38 for age.

**Decision Log for qualitative coding exercise**

**STAGE 1 (initial coding of 10% of items by authors VF and JGS and discussion of emerging codes)**

● Simple statements of “food” or similar (coffee, breakfast, etc.) are expressions of desires that are not temporally anchored (i.e., thought is not referring to time frame)

● “I need to ___” or similar is a statement of intent or a tentative plan, so it refers to something in the unspecified future, regardless of coder interpretation of when the event would logically take place.

● Statements of “food” or similar (coffee, breakfast, etc.) are referring to self-based thoughts unless otherwise explicitly mentioned

● “I’m still tired” or similar is a past unspecified occurrence, as it is something that was true in the past and is now continuing.

● “Time to get the children ready” or similar is about other, as it is a social action with “other” beneficiary

● “What time/day/etc is it” is temporal>other, as it is clearly a time anchored inquiry but cannot be said to be either future or past necessarily

● Any statement that explicitly refers to time but is not clearly related to future/past (i.e. “Why am I awake (it was 3am)” is coded as temporal>other, because it is anchored into time but not in relation to future/past

● Statements that are left blank or say “nothing” or “I can’t remember” are left completely blank

● References to being late are treated as future unspecified, as they refer to an individual thinking of an event that is yet to happen

● Statements of illness or physical discomfort are treated as negative valence

**STAGE 2 (coding of an additional 10% of data by VF & JGS, further discussion and collapsing of codes as necessary)**

● Statements of action that don't specify any time frame (i.e., "ringing my boyfriend") are no time reference, as we cannot suppose they are future

● to do list is things you either do or don't have to do, so "i have to work" and "i don't have to work" both apply

● "what things I have to do today" is establishing a to-do list, not establishing time

**Final codebook and dimension categorisation**

|  | **N1 code** | **N2 code** | **N3 code** | **N4 code** |
| --- | --- | --- | --- | --- |
| **Thought characterisation** | **Thoughts about feelings of states** | **(lack of) sleep or rest**  *Example: “I wish i (sic) slept more hours”* | | |
|  |  | **Dreams**  *Example: “…that was a weird dream”* | | |
|  |  | **Discomfort/sick/ill**  *Example: “I’m aching”* | | |
|  |  | **Waking up or being awoken** | Alarm clock, alarm or noise  *Example: “I need to turn my alarm off”* | |
|  |  |  | Being awake  *Example: “I don’t want to be awake yet.”* | |
|  |  |  | Being woken up  *Example: “why have the cats both woken me up earlier than usual?”* | |
|  | **Spatial or temporal orientation** | **Spatial orientation (inc. weather)**  *Example: “omg, what a bad weather”* | | |
|  |  | **Temporal orientation (day)** | What day is it  *Example: “What day is it”* | |
|  |  |  | How many days are left | |
|  |  |  | Known day  *Example: “ps5 is out today”* | |
|  |  | **Temporal orientation (time)** | What time is it  *Example: “What time is it?”* | |
|  |  |  | Known time  *Example: “Why am I awake (it was 3am)”* | |
|  |  |  | How much time is left/lateness  *Example: “Oh god I am late for work”* | |
|  | **Waking action** | **Immediate needs (water, food, bathroom)** | **Attending to bodily needs** | Drinking  *Example: “having a drink”* |
|  |  |  |  | Eating  *Example: “food”* |
|  |  |  |  | Medication  *Example: “Taking my medication for my chronic disease”* |
|  |  |  | **Getting up** | Showering  *Example: “Need to get a shower”* |
|  |  |  |  | Bathroom  *Example: “That I needed the bathroom”* |
|  |  |  |  | Toilet  *Example: “I need to go to the toilet”* |
|  |  |  |  | Getting out of bed  *Example: “Better get out of bed at some point”* |
|  |  |  |  | Getting ready or dressed  *Example: “I need to get ready for work”* |
|  |  | **Looking at technology (phone or email)**  *Example: “Check my phone.”* | | |
|  |  | **To-do list for the day** | **Establishing ‘to do list’ of the day**  *Example: “What I have to do in the day”* | |
|  |  |  | **Commitment to…** | Self (work/ chores/tasks)  *Example: “About my chores for the day”* |
|  |  |  |  | Other (work/ chores/tasks)  *Example: “Get kids ready for school”* |
|  |  |  |  |  |
| **Thought context** | **Dimension** | **Categories** | | |
|  | **Temporal window**  **(“when am I?”)** | **Thought is not referring to a timeframe** | | |
|  |  | **Thought refers to past** | Day  *Example: “Remembering something upsetting that happened to me yesterday.”* | |
|  |  |  | Week | |
|  |  |  | Unspecified  *Example: “something about the dream I was having, related to work”* | |
|  |  | **Thought refers to future** | Day  *Example: “work for the day”* | |
|  |  |  | Week  *Example: “About a hospital appointment next week.”* | |
|  |  |  | Unspecified  *Example: “how much work I needed to do”* | |
|  | **Question vs. statement** | **Question**  *Example: “what shall I wear?”* | | |
|  |  | **Statement**  *Example: “it’s dark”* | | |
|  | **Valence of statement** | **Positive**  *Example: “glad im (sic) here”* | | |
|  |  | **Negative**  *Example: “felt frustration and mild despair”* | | |
|  |  | **Neutral**  *Example: “To check my phone”* | | |
|  | **Protagonist** | **Self**  *Example: “I’m tired”* | | |
|  |  | **Other** | Partner  *Example: “Ringing and waking up my boyfriend”* | |
|  |  |  | Family/children/friends  *Example: “go and get my son”* | |
|  |  |  | iPhone/PS5/email/electronics  *Example: “I wonder if I’m going to have a lot of emails in my inbox”* | |

**S3: Numbers of reports in different code groups**


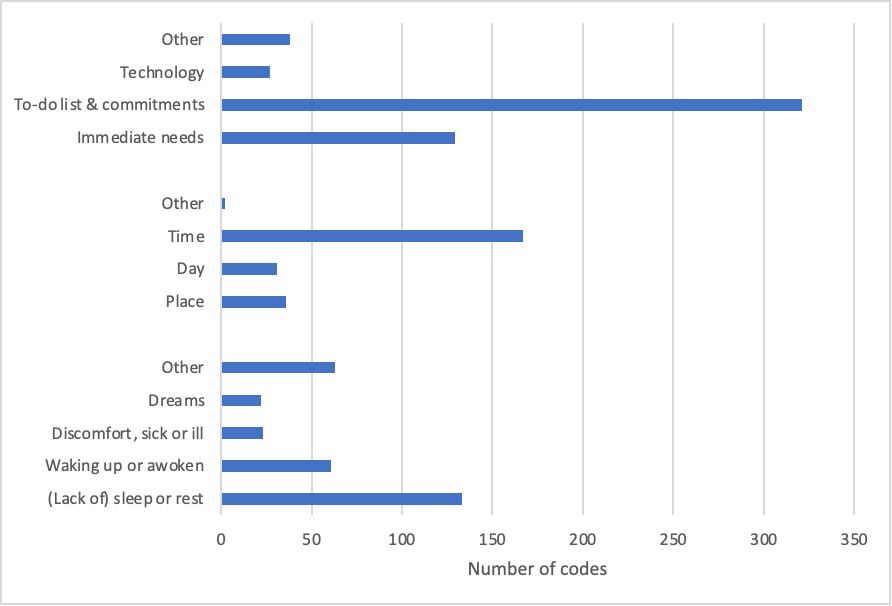


**S4: Pairwise comparisons for prior knowledge and dimension**

|  | **Establish Place** | **Establish Day** | **Establish Time** | **Know Place** | **Know Day** | **Know Time** |
| --- | --- | --- | --- | --- | --- | --- |
| **Establish Place** |  | -0.157* | -0.984** | -1.242** | -0.446** | 0.112 |
| **Establish Day** | 0.157* |  | -0.827** | -1.085** | -0.290** | 0.269** |
| **Establish Time** | 0.984** | 0.827** |  | -0.258** | 0.538** | 1.096** |
| **Know Place** | 1.242** | 1.085** | 0.258** |  | 0.796** | 0.796** |
| **Know Day** | 0.446** | 0.290** | -0.538** | -0.796** |  | 0.558** |
| **Know Time** | -0.112 | 0.269** | -1.096** | -1.354 | -0.558** |  |

***Note: boxes contain the mean difference between the values. *p < 0.05 level, **p < 0.001 level using Bonferroni correction.***

**S5: Pairwise comparisons for temporal orientation and temporal distance**

|  | **Day Ahead** | **Week Ahead** | **Year Ahead** | **Day Before** | **Week Before** | **Year Before** |
| --- | --- | --- | --- | --- | --- | --- |
| **Day Ahead** |  | 0.864** | 1.596** | 0.907** | 1.216** | 1.630** |
| **Week Ahead** | -0.864** |  | 0.732** | 0.044 | 0.352* | 0.766** |
| **Year Ahead** | -1.596** | -0.732** |  | -0.689** | -0.380** | 0.034 |
| **Day Before** | -0.907** | -0.044 | -1.596** |  | 0.308** | 0.722** |
| **Week Before** | -1.216** | -0.352** | 0.380** | -0.308** |  | 0.414** |
| **Year Before** | -1.630** | -0.766** | -0.034 | -0.722** | -0.414** |  |

***Note: boxes contain the mean difference between the values. *p < 0.05 level, **p < 0.001 level***

**References**

1. Blair E. A reflexive exploration of two qualitative data coding techniques. Journal of Methods and Measurement in the Social Sciences. 2015;6: 14–29.

2. King N. Template analysis. Qualitative methods and analysis in organizational research: A practical guide. Thousand Oaks, CA: Sage Publications Ltd; 1998. pp. 118–134.

3. O’Connor C, Joffe H. Intercoder reliability in qualitative research: debates and practical guidelines. International Journal of Qualitative Methods. 2020;19: 1609406919899220.

4. Strauss A, Corbin J. Discovery of grounded theory. 1967.

5. Williams M, Moser T. The art of coding and thematic exploration in qualitative research. International Management Review. 2019;15: 45–55.

6. Anderson C. Presenting and evaluating qualitative research. American journal of pharmaceutical education. 2010;74.

7. Burnard P. Writing a qualitative research report. Accident and emergency nursing. 2004;12: 176–181.
